# Supplementary material for: An integrative cross-omics analysis of DNA methylation sites of glucose and insulin homeostasis
Source: Nat Commun. 2019 Jun 13;10:2581. doi: 10.1038/s41467-019-10487-4 (PMC6565679; doi:10.1038/s41467-019-10487-4)
Supplement: Supplementary file 1 — Supplementary Information [file 41467_2019_10487_MOESM1_ESM.pdf]

An integrative cross-omics analysis of DNA methylation sites of glucose and insulin homeostasis

Liu et al

Supplementary Note 1: Supplementary Methods

Cohort-specific description - Discovery cohorts

Rotterdam study III-1 (RS III-1)

The Rotterdam Study (RS) is a large prospective, population-based cohort study in Rotterdam, the Netherlands, based on elderly aged 45 years and over living in the Ommoord district since 1990. The initial cohort, the Rotterdam Study I (RS-I) was started in 1989 and consisted of 7,983 subjects aged 55 years or over. In 1999, a second cohort named the Rotterdam Study II (RS-II) was started and consisted of 3,011 participants who had reached the age of 45 years since 1989 or who had moved into the study district. In 2006, the cohort was further extended with 3,932 subjects who were aged 45 years or over; the Rotterdam Study III (RS-III). Since the summer of 2016, another extension has started that includes all participants aged 40 years and over. The RS has been described in detail elsewhere[1]. The RS has been approved by the Medical Ethics Committee of the Erasmus Medical Center and by the review board of The Netherlands Ministry of Health, Welfare and Sports. All participants in the present analysis provided written informed consent to participate.

DNA methylation dataset was produced at the Genetic Laboratory (Department of Internal Medicine, Erasmus University Medical Center, Rotterdam, the Netherlands). The DNA methylation data were generated for a subset of 747 individuals of RS-III at baseline. DNA was extracted from whole peripheral blood by standardized salting out methods. This was followed by a bisulfide conversion using the Zymo EZ-96 DNA-methylation kit (Zymo Research, Irvine, CA, USA). The genome for each sample was then amplified, fragmented and hybridized to the Infinium Illumina HumanMethylation450 arrays according to the manufacturer's protocol. The quality control for samples was performed using the Methylation Module of the GenomeStudio software. Data were extracted into beta values from raw IDAT files. We excluded samples based on the detection p-value criteria >99% (n = 7), poor bisulfite conversion based on control dashboard check (n = 5) and failed chromosome X & Y clustering (n = 4). Data pre-processing was additionally performed using an R programming pipeline based on the pipeline developed by Tost & Touleimat[2], which includes additional parameters and options to pre-process and normalize methylation data directly from IDAT files. The beta values were extracted using methylumi[3]. We excluded probes which had a detection p-value >0.01 in >95% of samples, 11,648 probes at X and Y chromosomes were excluded to avoid gender bias. After filtering we ended up with 731 samples and 463,456 probes in the first dataset. The raw beta values were then background corrected and normalized using the DASEN option of the *WateRmelon* R-package[4].

The participants with more than eight hours overnight fasting were included. The blood was stored at −80°C in a number of 5-mL aliquots. Glucose levels were measured within 1 week of sampling using the glucose hexokinase method[5]. Plasma insulin was determined by metric assay (Biosource Diagnostics, Camarillo, CA). This assay has no cross-reactivity with either proinsulin or C-peptide. All measurements were done at the clinical chemistry laboratory at Erasmus Medical Center in Rotterdam.

The discovery sample consisted of 731 samples from which DNA methylation was available after quality control. Linear regression models for epigenome-wide association analyses, based on non-diabetic subjects and covariates available (i.e., age, sex, chip array number, position on the array, lymphocytes, monocytes, granulocytes and smoking in baseline model) were restricted to 626 fasting individuals that were used in the analysis.

Rotterdam study BIOS (RS-BIOS)

The Rotterdam Study has been described above.

DNA methylation was available from a second subset of 864 samples comprising individuals at their fifth, third and second visits of RS-II and RS-III respectively, between 2009-2013. Those individuals were not included in RSIII-1. DNA extraction, bisulfite sequencing and hybridization steps were performed following standard procedures described above, as well as pre-processing steps. In this dataset, outlying samples were checked using the first two principal components obtained by principal component analysis. None of the samples failed the quality control checks. We also identified potential sample mix-ups between genotype and normalized DNA methylation data using mix-up mapper[6]. No mix-ups were detected. After filtering as RSIII-1, we ended up with 767 samples and 419,937 probes in the second dataset. After exclusion criteria, we ended up with 705 samples in the analysis. The fasting glucose and insulin were measured in the same method as RSIII-1. The statistical analysis was linear regression which was the same as RSIII-1.

The Netherlands Twins Register (NTR)

The Netherlands Twin Register (NTR) was established in the late 1980s[7]. The current analyses include individuals who participated in the NTR biobank project[8]. The NTR study was approved by the Central Ethics Committee on Research Involving Human Subjects of the VU University Medical Center, Amsterdam, and all subjects provided written informed consent.

Blood sampling and DNA extraction procedures have been described in detail previously[8-10]. In short, venous blood samples were drawn between 07.00-11.00 am after an overnight fast and usually in the subjects' homes. DNA methylation was assessed with the Infinium HumanMethylation450 BeadChip Kit (Illumina, Inc.) as described previously[11]. 500ng of genomic DNA from whole blood was bisulfite treated using the Zymo Research 96-well plate using the standard protocol for Illumina 450k micro-arrays, by the Department of Molecular Epidemiology from the Leiden University Medical Center (LUMC), The Netherlands. Subsequent steps (i.e. sample hybridization, staining, scanning) were performed by the Erasmus Medical Center micro-array facility, Rotterdam, The Netherlands. Probes were set to missing in a sample if they had an intensity value of exactly zero, detection P-value > 0.01, or bead count < 3. Several checks were performed to confirm sample identity, by making use of previously collected genotype data, 65 SNP (control) probes targeted by the 450k array, and differential methylation patterns in males versus females. Intensity values were normalized with Functional Normalization[12] and normalized intensity values were converted into beta-values (β).

Analysis of DNA methylation included 3,004 methylation samples from 2,973 individuals (including 60 diabetes cases and 2,914 non-diabetic controls)[13]. For 31 non-diabetic control individuals, two longitudinal methylation samples (mean interval = 5 years) were included in this analysis. Subjects with methylation data were mostly twin pairs and a small group of parents, siblings and spouses of twins. Of the 3,004 samples, 2,843 samples were from individuals who fasted. The analysis of DNA methylation was performed on 2,753 non-diabetic individuals' data available.

Glucose and insulin were measured in blood plasma with the Vitros 250 Glucose assay (Johnson & Johnson, Rochester, USA) and the Immulite 1000 Insulin Method (Diagnostic Product Corporation, Los Angeles, USA), respectively[14].

DNA methylation analyses were performed using Generalized Estimating Equations with the R-package gee[15], which allows accounting for the correlations between family members and between repeated measures of the same individual. All analyses with continuous outcome measures were performed using the following gee model specifications: id=familynumber, family=gaussian, correlation structure=exchangeable. The following covariates were included in baseline model: sex, age,

percentages of neutrophils, monocytes, eosinophils, lymphocytes and basophils, row on the 450k array (continuous variable with values ranging from 1 to 6), 96-wells plate (factor variable with 34 levels) and smoking status.

The UK Adult Twin Registry (TwinsUK)

The TwinsUK cohort was established in 1992 to recruit monozygotic (MZ) and dizygotic (DZ) same-sex twins[16]. The majority of participants are healthy female Caucasians (age range from 16 to 98 years old). There are more than 13,000 twin participants from all regions across the United Kingdom and many have multiple visits over the years. The project was approved by the local Ethics Committee, and informed consent was obtained from all participants. The majority of TwinsUK epigenetic data used in this study are publicly available under GEO accession numbers GSE62992 and GSE121633. Additional individual-level data can be applied for through the TwinsUK data access committee. For information on access and how to apply please see http://twinsuk.ac.uk/resources-for-researchers/access-our-data/.

The Infinium HumanMethylation450 BeadChip (Illumina Inc, San Diego, CA) was used to measure DNA methylation. Details of the experimental approaches have been previously described[17]. To correct for technical issues caused by the two Illumina probe types, the beta mixture quantile dilation (BMIQ) method was performed[17]. The methylation data is also background corrected. DNA methylation probes that mapped incorrectly or to multiple locations in the reference sequence were removed. Probes with >1% subjects with detection *P*-value > 0.05 were also removed. Subjects with more than 5% missing probes were also removed.

Plasma samples from the participants with 10 hours of overnight fasting were measured for glucose and insulin levels. Glucose was measured by Enzymatic colorimetric slide assay (Johnson and Johnson Clinical Diagnostic Systems, Amersham, U.K.) and Ektachem 700 multichannel analyzer. Insulin was measured by Immunoassay (Abbott Laboratories, Maidenhead, U.K.). The methylation levels were collected in a total of 877 subjects. All phenotypes were collected and measured during the twins' clinical visits or were assessed by the self-report questionnaires. In total, 724 female Caucasian participants were included in the data analysis after data quality control exclusions. Association analysis was performed using linear mix model to account for family structure, and then used residuals to fit a linear model. Covariates in the regression model included batch effects (plate, position on the plate), age, smoking status, 6 blood cell count estimates (Houseman estimation method[18], including Granule cells, B-cell, eosinophils, lymphocytes, monocytes, and neutrophils)[18], family structure and zygosity structure. A linear mixed effect regression model was applied as the data contained MZ and DZ twins. Family structure and zygosity were included as random-effect terms, whilst all the other covariates were included as fixed-effect terms.

Cohort-specific description - Replication cohorts

Atherosclerosis Risk in Communities (ARIC) Study

The ARIC Study is a prospective cohort study of cardiovascular disease risk in four U.S. communities[19]. Between 1987 and 1989, 7,082 men and 8,710 women aged 45-64 years were recruited from Forsyth County, North Carolina; Jackson, Mississippi (African Americans only); suburban Minneapolis, Minnesota; and Washington County, Maryland. The ARIC Study protocol was approved by the institutional review board of each participating university. After written informed consent was obtained, including that for genetic studies, participants have completed a baseline clinical examination (Visit 1) and five subsequent follow-up exams (Visits 2-6). DNA samples for the methylation analysis presented here were collected at Visits 2 or 3.

Bisulfite conversion of 1 ug genomic DNA was performed using the EZ-96 DNA Methylation Kit (Deep Well Format) (Zymo Research; Irvine, CA, USA) according to the manufacturer's instructions (www.zymoresearch.com). Bisulfite-converted DNA was used for hybridization on the HumanMethylation450 BeadChip, following the Illumina Infinium HD Methylation protocol (www.illumina.com). The analysis of the ARIC data used Beta MIxture Quantile dilation (BMIQ)[17] to normalize type 2 design probes to approximate the statistical distribution of type 1 probes. Positive and negative controls and sample replicates were included on each 96-well plate assayed. After exclusion of controls, replicates, and samples with integrity issues or that failed bisulfite conversion, a total of 2,841 study participants had HumanMethylation450 data available for further quality control analyses. We removed poor-quality samples with pass rate <99%, that is, if the sample had at least 1% of CpG sites with detection p-value > 0.01 or missing, indicative of lower DNA quality or incomplete bisulfite conversion, samples with a possible gender mismatch based on evaluation of selected CpG sites on the Y chromosome, and participants with prevalent diabetes, missing data, or fasting < 8 hours, leaving a total of 1,875 samples available for analysis. The analysis was restricted to African Americans from the Mississippi and North Carolina centers.

Glucose was measured by Hexokinase assay on a Coulter DACOS (Coulter Instruments).

A mixed effects model was used in the replication with CpG as the dependent variable and fasting glucose as the independent variable. The covariates in the baseline model included age, sex, GWAS principal components, estimated white blood cell fractions by Houseman[18], chip and row (as random effects) and smoking status.

Baltimore Longitudinal Study of Aging (BLSA)

Begun in 1958, BLSA is a continuously enrolled cohort study of community-dwelling adults conducted by the National Institute on Aging Intramural Research Program (https://www.blsa.nih.gov). The study currently enrolls participants free of major chronic conditions and functional impairments. Participants return to the study clinic for follow-up visits at intervals of one to four years determined by age. The design, study population and measurement protocols of the BLSA have been previously described[20]. DNA methylation was assayed in DNA samples collected at visits between November 1993 and March 2010 from participants who also had genome-wide SNP array or concurrent neuroimaging data available: where only array data was available for a participant the most recent DNA sample was selected. Analyses included 142 non-diabetic participants of African ancestry and 402 of European ancestry with methylation data, complete covariates and ancestry information based on SNP array data. Study participants provided written informed consent and all BLSA study protocols are approved by the institutional review board of the National Institute of Environmental Health Sciences.

Genomic DNA was extracted from whole blood samples using the Gentra Puregene DNA purification system (Qiagen Inc., Germantown, MD) and quantified using Quant-iT Picogreen Reagent (Invitrogen-Thermo Fisher Scientific, Waltham, MA). Genomic DNA was bisulfite converted using the EZ DNA methylation kit (Zymo Research Corp., Irvine, CA) and DNA methylation was assayed using the Illumina Infinium HumanMethylation450 BeadChip (Illumina Inc., San Diego, CA) according to the manufacturer’s instructions. Prior to selection of the study sample for the present analyses quality filtering and normalization were performed across all BLSA DNA methylation samples. Samples identified as outliers by multi-dimensional scaling (> 3 standard deviations from the mean) or with high rates of single nucleotide polymorphism (SNP) mismatch (≥5 SNPs) were excluded. NOOB background correction and DASEN normalization were performed using the wateRmelon package[4]. Probes were excluded if the detection p-value was >.01 for >5% of samples.

In the BLSA fasting glucose and insulin were measured in plasma samples from the 0-minute time point of an oral glucose tolerance test after an overnight fast. Glucose was measured using a glucose oxidase analyzer (2006-2009: Beckman Instruments, Brea, CA; 2009-present: YSI Incorporated, Yellow Springs, OH). Insulin was measured using an enzyme-linked immunosorbent assay (Mercodia, Uppsala, Sweden).

Multivariable linear regression was used in the replication with Methylation β values as the dependent variable and fasting glucose or insulin as the independent variable. Covariates in the baseline model included age, sex, cell proportions (neutrophil, lymphocyte, monocyte, eosinophil), experimental batch and smoking status. This work utilized the computational resources of the NIH HPC Biowulf cluster (http://hpc.nih.gov).

Cardiovascular Health Study (CHS)

The CHS is a population-based cohort study of risk factors for coronary heart disease and stroke in adults ≥65 years conducted across four field centers[21]. The original predominantly European ancestry cohort of 5,201 persons was recruited in 1989-1990 from random samples of the Medicare eligibility lists; subsequently, an additional predominantly African-American cohort of 687 persons were enrolled for a total sample of 5,888. CHS was approved by institutional review committees at each field center and individuals in the present analysis had available DNA and gave informed consent including consent to use of genetic information for the study of cardiovascular disease. DNA methylation was measured on a randomly selected subset of 200 African Americans participants and 200 European descent participants from study year.

Methylation measurements were performed at the Institute for Translational Genomics and Population Sciences at the Harbor-UCLA Medical Center Institute for Translational Genomics and Population Sciences using the Infinium HumanMethylation450 BeadChip (Illumina Inc., San Diego, CA). Quality control was performed in the *minfi* R-package[12,22,23]. (version 1.12.0, http://www.bioconductor.org/packages/release/bioc/html/minfi.html). Samples with low median intensities of below 10.5 (log2) across the methylated and unmethylated channels, samples with a proportion of probes falling detection of greater than 0.5%, samples with QC probes falling greater than 3 standard deviations from the mean, sex check mismatches, or failed concordance with prior genotyping were removed. Methylation values were normalized using the Subset-quantile Within Array Normalization (SWAN) quantile normalization method[23]. Since white blood cell proportions were not directly measured in CHS they were estimated from the methylation data using the Houseman method[18].

The serum from participants after 12 hours of overnight fasting was measured in glucose and insulin. Glucose was measured by Kodak Ektachem 700 Analyzer. Insulin was measured by Kodak Ektachem 700 Analyzer.

It ended up with 160 non-diabetic participants from European ancestry and 147 from American African ancestry who have both qualified methylation data and phenotype data. Linear regression was used in the replication with CpG as the dependent variable and fasting glucose or insulin as the independent variable. The covariates in the baseline model included age, sex, 2 genetic principal components, estimated cell counts by Houseman[18], chip number, column and row and smoking status.

Framingham Heart Study Cohort (FHS)

The Framingham Heart Study (FHS)[24] is a large-scale longitudinal study started in 1948, initially investigating the common factors of characteristics that contribute to cardiovascular disease (CVD), https://www.framinghamheartstudy.org/index.php. The study at first enrolled participants living in the town of Framingham, Massachusetts, who were free of overt symptoms of CVD, heart attack or stroke at enrollment. In 1971, the study started FHS Offspring Cohort to enroll a second generation of the original participants' adult children and their spouses (n= 5,124) for conducting similar examinations[25]. Participants

from the FHS Offspring Cohort were eligible for our study if they attended the eighth examination cycle (2005-2008) and consented to have their DNA used for genetic research. All participants provided written informed consent at the time of each examination visit. The study protocol was approved by the Institutional Review Board at Boston University Medical Campus (Boston, MA). The FHS data are available in dbGaP (accession number: phs000342.v17.p10 and phs000724.v2.p9).

Peripheral blood samples were collected at the 8th examination. The EZ DNA Methylation Kit (Zymo Research Corporation) was used for bisulfite conversion. DNA methylation quantification was conducted in two laboratory batches using the Illumina Infinium HumanMethylation450 array (Illumina). Methylation beta values were generated using the Bioconductor *minfi* package with background correction[22]. For sample quality control, we excluded samples with missing rate > 1% at detection P-value <0.01, poor matching to the single nucleotide polymorphism (SNP) control probe locations, and outliers by multi-dimensional scaling techniques. These procedures left 2,377 participants with phenotype data (n=442 for batch 1 and n=1,935 for batch 2) and 443,312 probes for analyses. In total, 2,157 non-diabetic participants were available for both DNA methylation, phenotype and covariate data, used in the current study. The raw beta values were then background corrected and normalized using the DASEN option of the WateRmelon R-package[4].

The plasma from participants with more than eight hours overnight fasting was used to measure glucose and insulin. Glucose was measured by Hexokinase (Glucose/HK, Riche Hitachi 911, Roche Diagnostics)[5]. Insulin was measured by Immunoassay (Roche Diagnostics).

Linear mixed effects model was used in the replication with DNA methylation as the dependent variable and fasting glucose or insulin as the independent variable. The covariates in the baseline model included batch effects (lab, plate, column, row), Houseman imputed cell counts[18], age, sex, and smoking, with a random effect to account for family structure.

The Genetic Epidemiology Network of Arteriopathy (GENOA)

The Genetic Epidemiology Network of Arteiopathy (GENOA) study is a community-based study of hypertensive sibships that was designed to investigate the genetics of hypertension and target organ damage in African Americans from Jackson, Mississippi and non-Hispanic whites from Rochester, Minnesota[26]. In the initial phase of the GENOA study (Phase I: 1996-2001), all members of sibships containing ≥ 2 individuals with essential hypertension clinically diagnosed before age 60 were invited to participate, including both hypertensive and normotensive siblings. Exclusion criteria of the GENOA study were secondary hypertension, alcoholism or drug abuse, pregnancy, insulin-dependent diabetes mellitus, or active malignancy. Eighty percent of African Americans (1,482 subjects) and 75% of non-Hispanic whites (1,213 subjects) from the initial study population returned for the second examination (Phase II: 2001-2005 Written informed consent was obtained from all subjects and approval was granted by participating institutional review boards.

DNA methylation was measured on the peripheral blood leukocytes. The EZ DNA Methylation Gold Kit (Zymo Research, Irvine CA) was used for bisulfite conversion. The methylation assay was performed at the Mayo Clinic Advanced Genomics Technology Center using Illumina® Infinium HumanMethylation450 BeadChip. The SWAN in *minfi* R-package was used for background adjustment, color balance adjustment, and quantile normalization[22,23]. Cell proportions for CD8 T lymphocytes, CD4 T lymphocytes, natural killer cells, B cells, monocytes, and granulocytes were estimated using Houseman's method[18]. Detection p-value was calculated for each site and beta values were set to missing if a site had detection p-value>0.01. In all samples, > 95% of probes had a detection P-value<0.01; thus, no samples were excluded from analysis.

Study visits were made in the morning after an overnight fast of at least eight hours. The serum was measured in glucose and insulin. Glucose was measured by Hexokinase reagent from Boehringer Mannheim (Indianapolis, IN 46256) on a Hitachi 911 Chemistry Analyzer (Roche Diagnostics, Indianapolis, IN). Insulin was measured by two-site immunoenzymatic assay performed on the Access automated immunoassay system (Beckman Instruments, Chaska, MN).

It ended up with 268 non-diabetic participants from American African ancestry who have both qualified methylation data and phenotype data. Linear mixed effects model with *lmer* function in lme4 R-package was used in the replication with CpG as the dependent variable and fasting glucose or insulin as the independent variable. The covariates in the baseline model included age, sex, cell proportions (estimated using Houseman's method[18]) and batch effects (plate, row, and column as random effects).

Genetics of Lipid Lowering Drugs and Diet Network (GOLDN)

The Genetics of Lipid Lowering Drugs and Diet Network (GOLDN) study, described in detail in prior publications[27], recruited families with at least two siblings from the participants of the National Heart, Lung, and Blood Institute Family Heart Study at the Minneapolis and Salt Lake City sites. The study protocol was approved by the Institutional Review Boards at the University of Minnesota, University of Utah, and Tufts University/New England Medical Center, and written informed consent was obtained from all participants.

CD4+ T-cells were isolated from frozen buffy coat samples isolated from peripheral blood collected at the baseline visit (prior to intervention). DNA was extracted using DNeasy kits (Qiagen, Venlo, Netherlands). 500ng of each DNA sample was treated with sodium bisulfite (Zymo Research, Irvine, CA) and methylation was measured using the Infinium HumanMethylation450 array (Illumina, San Diego, CA) as previously described. Samples with more than 1.5% missing data points across ~470,000 autosomal CpGs were excluded. The filtered β scores were normalized using the ComBat R-package[28]. Normalization was performed on random subsets of 10,000 CpGs per run, with each array of 12 samples used as a batch. Probes from the Infinium I and II chemistries were separately normalized and β scores for Infinium II probes were then adjusted using the equation derived from fitting a second order polynomial to the observed methylation values across all pairs of probes located <50bp apart (within-chemistry correlations >0.99), where one probe was Infinium I and one was Infinium II.

The plasma from participants after overnight fasting was measured in glucose and insulin. Glucose was measured by the hexokinase-mediated reaction on the Hitachi commercial kit (Roche Diagnostics). Insulin was measured by a commercial kit using competitive radioimmunoassay (Linco Research, St. Charles, MO).

It ended up with 917 non-diabetic participants from European ancestry who have both qualified methylation data and phenotype data. Linear mixed effect model was used in the replication with CpG as the dependent variable and fasting glucose or insulin as the independent variable. The covariates in the baseline model included age, sex, cell proportions (neutrophil, lymphocyte, monocyte, eosinophil), experimental batch and smoking status.

Hypertension Genetic Epidemiology Network (HyperGEN)

The Hypertension Genetic Epidemiology Network (HyperGEN) study is a cross-sectional, population-based study of 1,224 hypertensive African-American sibships initially recruited between 1996 and 1999. The study was later extended to other siblings and offspring of the original sib-pair. Using stored samples, an ancillary epigenetic study was conducted among cohort participants belonging to the highest and lowest quartiles (total N = 636) of left ventricular mass[29]. The study protocols and the process for obtaining informed consent were approved by the Institutional Review Committees at the local field centers.

The Illumina HumanMethylation450 array was used to analyze DNA extracted from buffy coat. These arrays can measure DNA methylation at > 480,000 CpG sites. Briefly, 500 ng of buffy coat DNA was hybridized to the Methyl450 array after bisulfite conversion with EZ DNA kits (Zymo Research, Irvine, CA) prior to standard Illumina amplification, hybridization, and imaging steps. The resulting intensity files were analyzed with Illumina's GenomeStudio, which generated beta (β) scores (i.e., the proportion of total signal from the methylation-specific probe or color channel) and detection p values. Quality control measures were conducted by removing samples having more than 1% of CpG sites with a detection p-value > 0.05, and CpG sites with detection p-value > 0.01 set as missing. After these filters, 484,366 CpG sites were eligible for analysis. We normalized the β scores using SWAN method in *minfi* R-package to correct for differences between batches and the type I and type II assay designs within a single 450k array[22,23]. Cell count proportions (CD8 T lymphocytes, CD4 T lymphocytes, natural killer cells, B cells, and monocytes) were created using the algorithm developed by Houseman[18], which predicts the underlying cellular composition of each sample from DNA methylation patterns.

The plasma from participants after overnight fasting was measured in glucose and insulin. Glucose was measured by using Elan Glucose reagent (hexokinase method). Insulin was measured by automated immunoassay instrument and its ultra-sensitive insulin kit (Beckman Coulter, Fullerton, CA, USA). It ended up with 469 non-diabetic participants from American African ancestry who have both qualified methylation data and phenotype data. Linear mixed effect model was used in the replication with CpG as the dependent variable and fasting glucose or insulin as the independent variable. The covariates in the baseline model included age, sex, cell proportions (CD8 T lymphocytes, CD4 T lymphocytes, natural killer cells, B cells, and monocytes), experimental batch and smoking status.

Invecchiare in Chianti Study (InCHIANTI)

The InCHIANTI is a population-based prospective cohort study of residents from two areas in the Chianti region of Tuscany, Italy (http://inchiantistudy.net/wp). Study participants were enrolled between 1998 and 2000 and have subsequently been followed at three-year intervals. Selection of study participants and data collection procedures have been previously described[30]. DNA methylation was assayed in DNA samples corresponding to participants with sufficient DNA at both the baseline (1998-2000) and 9-year follow-up (2007-2009) visits. Analyses included 433 non-diabetic European participants with methylation data and complete covariates. InCHIANTI protocols were approved by the Instituto Nazionale Riposo e Cura Anziani institutional review board in Italy and study participants provided informed consent.

Genomic DNA was extracted from buffy coat samples using an AutoGen Flex and quantified on a Nanodrop1000 spectrophotometer. Genomic DNA was bisulfite converted (Zymo EZ-96 DNA Methylation Kit - Zymo Research Corp., Irvine, CA) and DNA methylation was assayed using the Illumina Infinium HumanMethylation450 BeadChip (Illumina Inc., San Diego, CA) as previously described[31]. Prior to selection of the study sample for the present analyses quality filtering and normalization were performed across all InCHIANTI DNA methylation samples (baseline and 9-year follow-up). Samples identified as outliers by multi-dimensional scaling (>3 standard deviations from the mean) or with high rates of single nucleotide polymorphism (SNP) mismatch (≥5 SNPs) were excluded. NOOB background correction and DASEN normalization were performed using the wateRmelon package[4]. Probes were excluded if the detection p-value was >0.01 for >5% of samples.

At the InCHIANTI baseline study visit, blood was drawn using a standardized protocol after an overnight fast. Fasting glucose was measured in serum using an enzymatic colorimetric assay (Roche-Hitachi 917 analyzer - Roche Diagnostics, Mannheim, Germany). Fasting insulin was measured in EDTA plasma using a commercial double-antibody, solid-phase radioimmunoassay (Sorin Biomedica, Milan, Italy).

Multivariable linear regression was used in the replication with Methylation β values as the dependent variable and fasting glucose or insulin as the independent variable. Covariates in the baseline model included age, sex, cell proportions (neutrophil, lymphocyte, monocyte, eosinophil), experimental batch and smoking status. This work utilized the computational resources of the NIH HPC Biowulf cluster (http://hpc.nih.gov).

Kooperative Gesundheitsforschung in der Region Augsburg (KORA)

The Cooperative Health Research in the Region of Augsburg (KORA) study is a series of independent surveys and follow-up studies from the general population living in the region of Augsburg, Southern Germany. All participants are residents of German nationality identified through the local registration office. The study was approved by the ethics committee of the Bavarian Medical Association, and informed written consent was obtained from all participants[32].

For the present study, blood was collected in fasting subjects of the KORA F4 subjects without stasis and kept at 4°C until centrifugation. DNA methylation measurements were performed on whole blood samples of the KORA F4 study using Illumina HumanMethylation450 BeadChip arrays and data were preprocessed as described previously[33,34]. Briefly, genomic DNA was bisulfite converted, amplified and enzymatically fragmented. After application of samples, the arrays were fluorescently stained and scanned using Illumina HiScan SQ scanner. Raw methylation values were pre-processed using R, version 3.0.1, partly following a pipeline developed by Touleimat and Tost[2]. β-values set to missing if detection p-value $\geq$ 0.01 or beads $\leq$ 3. Samples were excluded if detection rate < 95%. Color bias adjustment and background correction based on negative control probes were conducted (R-package *lumi*, version 2.12.0)[35]. Finally, beta-mixture quantile normalization (BMIQ)[36] was applied to the DNA methylation data (R-package WateRmelon, version 1.0.3).

Fasting venous blood was collected with minimal stasis, centrifuged, refrigerated at 4 to 8°C, and analyzed in the central laboratory within a maximum of 6 hours. Glucose was measured by Hexokinase method[5] (Glucoquant). Insulin was measured by ELISA (Invitrogen, Karlsruhe, Germany).

DNA methylation data were available in 1,799 subjects. For the current analysis, a total of 1,488 individuals with phenotype, DNA methylation data and covariates available were included in the present analyses. Linear regression was used in the replication with CpG as the dependent variable and fasting glucose or insulin as the independent variable. The covariates in the baseline model included age, sex, the first 20 principal components, cell proportions (estimated using Houseman's method[18]: CD8 T lymphocytes, CD4 T lymphocytes, natural killer cells, B cells, and monocytes) and smoking status.

Women's Health Initiative - Broad Agency Award 23 (WHI-BAA23)

WHI study is a national study that began in 1993 which enrolled postmenopausal women between the ages of 50-79 years into either randomized clinical trials (RCTs), http://www.nhlbi.nih.gov/whi/. We included WHI participants available for both phenotype and DNA methylation array data including 1,658 non-diabetic women from the study BAA23. The study focuses on identifying miRNA and genomic biomarkers of congenital heart disease (CHD), integrating the biomarkers into diagnostic and prognostic predictors of CHD and other related phenotypes, and other objectives can be found online[37]. The study populations are across three sub WHI cohorts including (a) GARNET, (b) WHIMS and (c) SHARe. The protocol and consent

forms were approved by the institutional review boards of all participating institutions and all women provided written informed consent.

DNA methylation quantification: in brief, bisulfite conversion using the Zymo EZ DNA Methylation Kit (Zymo Research, Orange, CA, USA) as well as subsequent hybridization of the HumanMethylation450 BeadChip (Illumina, San Diego, CA), and scanning (iScan, Illumina) were performed according to the manufacturers protocols by applying standard settings. DNA methylation levels (β values) were determined by calculating the ratio of intensities between methylated (signal A) and unmethylated (signal B) sites. Specifically, the β value was calculated from the intensity of the methylated (M corresponding to signal A) and unmethylated (U corresponding to signal B) sites, as the ratio of fluorescent signals $\beta = Max(M,0)/[Max(M,0)+Max(U,0)+100]$. Thus, β values range from 0 (completely unmethylated) to 1 (completely methylated). The serum from participants with more than eight hours overnight fasting was measured in glucose and insulin. Glucose was measured with Hexokinase method[5] on the Hitachi 747 (Boehringer Mannheim Diagnostics, Indianapolis, Indiana). Insulin was measured by ELISA, immunoassay kits from Diagnostic Systems Laboratories (DSL; Webster, Texas).

DNA methylation data were normalized using BMIQ[36], then stage- and plate-adjusted using ComBat[28]. Linear mixed effects model was used in the replication with CpG as the dependent variable and fasting glucose or insulin as the independent variable. The covariates in the baseline model included batch effects (lab, plate, column, row) and Houseman imputed cell counts[18], and age, sex, family structure and smoking.

Women's Health Initiative - Epigenetic Mechanisms of PM-Mediated CVD (WHI-EMPC)

WHI-EMPC is an ancillary study of epigenetic mechanisms underlying associations between ambient particulate matter (PM) air pollution and cardiovascular disease (CVD) in the Women's Health Initiative clinical trials (CT) cohort. The detail of the project information can be found online[38]. The WHI-EMPC study population is a stratified, random sample of 2,200 WHI CT participants who were examined between 1993 and 2001; had available buffy coat, core analytes, electrocardiograms, and ambient concentrations of PM; but were not taking anti-arrhythmic medications at the time. The protocol and consent forms were approved by the institutional review boards of all participating institutions and all women provided written informed consent.

As such, WHI-EMPC is representative of the larger, multiethnic WHI CT population from which it was sampled: n = 68,132 participants aged 50-79 years who were randomized to hormone therapy, calcium/vitamin D supplementation, and/or dietary modification in 40 U.S. clinical centers at the baseline exam (1993-1998) and re-examined in the fasting state one, three, six, and nine years later[39,40].

Current analyses involved information collected at the first available visit with available DNA methylation data and stratification by race/ethnicity (black; Hispanic/Latino; white). We included the following WHI participants with phenotype and DNA methylation array data: 1,634 non-diabetic women from the Epigenetic Mechanisms of PM-Mediated CVD Risk (WHI-EMPC). Genome-wide DNA methylation at CpG sites was measured using the Illumina 450K Infinium Methylation BeadChip, quantitatively represented by beta (the percentage of methylated cytosines over the sum of methylated and unmethylated cytosines), and quality controlled using the following filters: detection p-values > 0.01 in > 10% of samples and detection p-values > 0.01 or missing in > 1% of probes, yielding values of beta at 484,220 sites. Beta-mixture quantile normalization was implemented using BMIQcite[17] and empirical Bayes methods of batch adjustment for stage and plate were implemented in ComBat[28].

Serum from participants fasting > 8 hours was assayed for glucose and insulin. Glucose was assayed by the Hexokinase method[5] on the Hitachi 747 (Boehringer Mannheim Diagnostics, Indianapolis, Indiana). Serum insulin was measured by radioimmunoassay or stepwise sandwich enzyme-linked immunosorbent assay. Results of the two methods were comparable at insulin concentrations < 60 uIU/ml. Moreover, the intra-class correlation coefficient among 564 WHI participants with repeatedly measured insulin (uIU/mL) obtained 8-15 weeks apart was 0.71[41].

Linear mixed-effects model was used in the replication with CpG site-specific DNA methylation as the dependent variable and fasting glucose or insulin as the independent variable. Modeled epigenome-wide associations also adjusted for age, sex, smoking status, estimated cell subtype proportions (CD8-T, CD4-T, B cell, natural killer, monocyte, and granulocyte)[18], ancestry using principal components 1-10, and technical covariates including chip, row and column.

References

1 Ikram, M. A. *et al.* The Rotterdam Study: 2018 update on objectives, design and main results. *Eur J Epidemiol* **32**, 807-850, (2018).

2 Touleimat, N. & Tost, J. Complete pipeline for Infinium((R)) Human Methylation 450K BeadChip data processing using subset quantile normalization for accurate DNA methylation estimation. *Epigenomics* **4**, 325-341 (2012).

3 Davis S, D. P., Bilke S, Triche T, Jr. and Bootwalla M. methylumi: Handle Illumina methylation data. *R package version 2.14.0* (2015).

4 Pidsley, R. *et al.* A data-driven approach to preprocessing Illumina 450K methylation array data. *BMC Genomics* **14**, 293 (2013).

5 Neeley, W. E. Simple automated determination of serum or plasma glucose by a hexokinase-glucose-6 -phosphate dehydrogenase method. *Clin Chem* **18**, 509-515 (1972).

6 Westra, H. J. *et al.* MixupMapper: correcting sample mix-ups in genome-wide datasets increases power to detect small genetic effects. *Bioinformatics* **27**, 2104-2111 (2011).

7 Boomsma, D. I. *et al.* Netherlands Twin Register: from twins to twin families. *Twin Res Hum Genet* **9**, 849-857, (2006).

8 Willemsen, G. *et al.* The Netherlands Twin Register biobank: a resource for genetic epidemiological studies. *Twin Res Hum Genet* **13**, 231-245, (2010).

9 Wright, F. A. *et al.* Heritability and genomics of gene expression in peripheral blood. *Nat Genet* **46**, 430-437, (2014).

10 Jansen, R. *et al.* Sex differences in the human peripheral blood transcriptome. *BMC Genomics* **15**, 33, (2014).

11 van Dongen, J. *et al.* Genetic and environmental influences interact with age and sex in shaping the human methylome. *Nat Commun* **7**, 11115, (2016).

12 Fortin, J. P. *et al.* Functional normalization of 450k methylation array data improves replication in large cancer studies. *Genome Biol* **15**, 503, (2014).

13 Willemsen, G. *et al.* The Concordance and Heritability of Type 2 Diabetes in 34,166 Twin Pairs From International Twin Registers: The Discordant Twin (DISCOTWIN) Consortium. *Twin Res Hum Genet* **18**, 762-771, (2015).

14 van Dongen, J., Willemsen, G., Chen, W. M., de Geus, E. J. & Boomsma, D. I. Heritability of metabolic syndrome traits in a large population-based sample. *J Lipid Res* **54**, 2914-2923, (2013).

15 Zeger, S. L. & Liang, K. Y. Longitudinal data analysis for discrete and continuous outcomes. *Biometrics* **42**, 121-130 (1986).
16 Moayyeri, A., Hammond, C. J., Hart, D. J. & Spector, T. D. The UK Adult Twin Registry (TwinsUK Resource). *Twin Res Hum Genet* **16**, 144-149 (2013).
17 Teschendorff, A. E. *et al.* A beta-mixture quantile normalization method for correcting probe design bias in Illumina Infinium 450 k DNA methylation data. *Bioinformatics* **29**, 189-196 (2013).
18 Houseman, E. A. *et al.* DNA methylation arrays as surrogate measures of cell mixture distribution. *BMC Bioinformatics* **13**, 86, (2012).
19 The Atherosclerosis Risk in Communities (ARIC) Study: design and objectives. The ARIC investigators. *Am J Epidemiol* **129**, 687-702 (1989).
20 Shock, N. W. Normal human aging: The Baltimore longitudinal study of aging. (1984).
21 Fried, L. P. *et al.* The Cardiovascular Health Study: design and rationale. *Ann Epidemiol* **1**, 263-276, (1991).
22 Aryee, M. J. *et al.* Minfi: a flexible and comprehensive Bioconductor package for the analysis of Infinium DNA methylation microarrays. *Bioinformatics* **30**, 1363-1369, (2014).
23 Maksimovic, J., Gordon, L. & Oshlack, A. SWAN: Subset-quantile within array normalization for illumina infinium HumanMethylation450 BeadChips. *Genome Biol* **13**, R44, (2012).
24 Dawber, T. R., Meadors, G. F. & Moore, F. E., Jr. Epidemiological approaches to heart disease: the Framingham Study. *Am J Public Health Nations Health* **41**, 279-281 (1951).
25 Kannel, W. B., Feinleib, M., McNamara, P. M., Garrison, R. J. & Castelli, W. P. An investigation of coronary heart disease in families. The Framingham offspring study. *Am J Epidemiol* **110**, 281-290 (1979).
26 Daniels, P. R. *et al.* Familial aggregation of hypertension treatment and control in the Genetic Epidemiology Network of Arteriopathy (GENOA) study. *Am J Med* **116**, 676-681, (2004).
27 Lai, C. Q. *et al.* Fenofibrate effect on triglyceride and postprandial response of apolipoprotein A5 variants: the GOLDN study. *Arterioscler Thromb Vasc Biol* **27**, 1417-1425, (2007).
28 Johnson, W. E., Li, C. & Rabinovic, A. Adjusting batch effects in microarray expression data using empirical Bayes methods. *Biostatistics* **8**, 118-127, (2007).
29 Akinyemiju, T. *et al.* Epigenome-wide association study of metabolic syndrome in African-American adults. *Clin Epigenetics* **10**, 49, (2018).
30 Ferrucci, L. *et al.* Subsystems contributing to the decline in ability to walk: bridging the gap between epidemiology and geriatric practice in the InCHIANTI study. *Journal of the American Geriatrics Society* **48**, 1618-1625 (2000).
31 Gibbs, J. R. *et al.* Abundant quantitative trait loci exist for DNA methylation and gene expression in human brain. *PLoS genetics* **6**, e1000952 (2010).
32 Wichmann, H. E., Gieger, C., Illig, T. & Group, M. K. S. KORA-gen--resource for population genetics, controls and a broad spectrum of disease phenotypes. *Gesundheitswesen* **67 Suppl 1**, S26-30, (2005).
33 Wahl, S. *et al.* On the potential of models for location and scale for genome-wide DNA methylation data. *BMC Bioinformatics* **15**, 232, (2014).

34 Pfeiffer, L. *et al.* DNA methylation of lipid-related genes affects blood lipid levels. *Circ Cardiovasc Genet* **8**, 334-342, (2015).

35 Du, P., Kibbe, W. A. & Lin, S. M. lumi: a pipeline for processing Illumina microarray. *Bioinformatics* **24**, 1547-1548, (2008).

36 Teschendorff, A. E. *et al.* Epigenetic variability in cells of normal cytology is associated with the risk of future morphological transformation. *Genome Med* **4**, 24, (2012).

37 BA23. BA23 - Integrative genomics and risk of CHD and related phenotypes in the Women's Health Initiative. *Available at https://www.whi.org/researchers/data/WHIStudies/StudySites/BA23/Pages/home.aspx*.

38 Whitsel, E. Epigenetic Mechanisms of PM-Mediated CVD Risk (WHI-EMPC; R01-ES020836). *National Institutes of Health. U.S. Department of Health and Human Services. Research Portfolio Online Reporting Tools* [**https://projectreporter.nih.gov/project_info_description.cfm?aid=9054857&icde=40451534&ddparam=&ddvalue=&ddsub=&cr=1&csb=default&cs=ASC&pball]** and [**https://projectreporter.nih.gov/project_info_description.cfm?aid=8369149&icde=27485446**]

39 Design of the Women's Health Initiative clinical trial and observational study. The Women's Health Initiative Study Group. *Control Clin Trials* **19**, 61-109, (1998).

40 Anderson, G. L. *et al.* Implementation of the Women's Health Initiative study design. *Ann Epidemiol* **13**, S5-17, (2003).

41 Langer, R. D. *et al.* The Women's Health Initiative Observational Study: baseline characteristics of participants and reliability of baseline measures. *Ann Epidemiol* **13**, S107-121(2003).

Supplementary Note 2: Cohort-specific acknowledgments

RS: The generation and management of the Illumina 450K methylation array data (EWAS data) and the Illumina exome chip v1.0 array data for the Rotterdam Study were executed by the Human Genotyping Facility of the Genetic Laboratory of the Department of Internal Medicine, Erasmus MC, Rotterdam, The Netherlands. We thank Michael Verbiest, Mila Jhamai, Sarah Higgins, Marijn Verkerk and Lisette Stolk for their help in creating the methylation database. We would like to acknowledge members of the BIOS consortium (https://www.bbmri.nl/?p=259). The Rotterdam Study is funded by Erasmus Medical Centre and Erasmus University, Rotterdam, Netherlands Organization for the Health Research and Development (ZonMw), the Research Institute for Diseases in the Elderly (RIDE), the Ministry of Education, Culture and Science, the Ministry for Health, Welfare and Sports, the European Commission (DG XII), and the Municipality of Rotterdam. The EWAS data was funded by the Genetic Laboratory of the Department of Internal Medicine, Erasmus MC, and by the Netherlands Organization for Scientific Research (NWO; project number 184021007). This project received funding from the European Union's Horizon 2020 research and innovation programme (733206, LIFECYCLE). L.D. received funding from the co-funded programme ERA-Net on Biomarkers for Nutrition and Health (ERA HDHL) (ALPHABET project, Horizon 2020 (grant agreement no 696295; 2017), ZonMW The Netherlands (no 529051014; 2017)). Epigenetic and transcriptomics have been funded by Biobanking and Biomolecular Resources Research Infrastructure (BBMRI)–NL (184.021.007).

NTR: NTR funding was obtained from the Netherlands Organization for Scientific Research (NWO, NWO-Groot 480-15-001/674 and Middelgroot-911-09-032), Biobanking and Biomolecular Resources Research Infrastructure (BBMRI–NL, 184.021.007 and 184.033.111), Center for Medical Systems Biology (CSMB, NWO Genomics), VU University's Institute for Health and Care Research (EMGO+) and Neuroscience Campus Amsterdam (NCA), the European Research Council (ERC Advanced, 230374), Rutgers University Cell and DNA Repository (NIMH U24 MH068457-06), the Avera Institute, Sioux Falls, South Dakota (USA) and the National Institutes of Health (NIH), Grand Opportunity grants 1RC2 MH089951 and 1RC2 MH089995).

TwinsUK: The TwinsUK study was funded by the Wellcome Trust; European Community's Seventh Framework Programme (FP7/2007–2013); National Institute for Health Research (NIHR)- funded BioResource, Clinical Research Facility and Biomedical Research Centre based at Guy's and St Thomas' NHS Foundation Trust in partnership with King's College London. SNP genotyping was performed by The Wellcome Trust Sanger Institute and National Eye Institute via NIH/CIDR. The TwinsUK epigenetic study received further support from the ESRC (ES/N000404/1 to J.T.B).

ARIC: The Atherosclerosis Risk in Communities study has been funded in whole or in part with Federal funds from the National Heart, Lung, and Blood Institute, National Institutes of Health, Department of Health and Human Services (contract numbers HHSN268201100005C, HHSN268201100006C, HHSN268201100007C, HHSN268201100008C, HHSN268201100009C, HHSN268201100010C, HHSN268201100011C, HHSN268201100012C, HHSN268201700001I, HHSN268201700002I, HHSN268201700003I, HHSN268201700004I and HHSN268201700005I). The authors thank the staff and participants of the ARIC study for their important contributions. Funding support for 'Building on GWAS for NHLBI-diseases: the U.S. CHARGE consortium' was provided by the National Institutes of Health (NIH) through the American Recovery and Reinvestment Act of 2009 (ARRA) (5RC2HL102419). Funding was also supported by R01NS087541.

BLSA: The BLSA is supported by the Intramural Research Program of the NIH, National Institute on Aging. Analytic work described here was supported by the Intramural Research Program of the NIH, National Institute on Aging.
CHS: Infrastructure for the CHARGE Consortium is supported in part by the National Heart, Lung, and Blood Institute grant R01HL105756. The CHS research was supported by NHLBI contracts HHSN268201200036C, HHSN268200800007C, HHSN268201800001C, N01HC55222, N01HC85079, N01HC85080, N01HC85081, N01HC85082, N01HC85083, N01HC85086; and NHLBI grants U01HL080295, U01HL130114, K08HL116640, R01HL087652, R01HL092111, R01HL103612, R01HL105756, R01HL103612, R01HL111089, R01HL116747 and R01HL120393 with additional contribution from the National Institute of Neurological Disorders and Stroke (NINDS). Additional support was provided through R01AG023629 from the National Institute on Aging (NIA), Merck Foundation / Society of Epidemiologic Research as well as Laughlin Family, Alpha Phi Foundation, and Locke Charitable Foundation. A full list of principal CHS investigators and institutions can be found at CHS-NHLBI.org. The provision of genotyping data was supported in part by the National Center for Advancing Translational Sciences, CTSI grant UL1TR000124, and the National Institute of Diabetes and Digestive and Kidney Disease Diabetes Research Center (DRC) grant DK063491 to the Southern California Diabetes Endocrinology Research Center.
FHS: The Framingham Heart Study is funded by National Institutes of Health contract N01-HC-25195 and HHSN268201500001I. The laboratory work for this investigation was funded by the Division of Intramural Research, National Heart, Lung, and Blood Institute, National Institutes of Health, Bethesda, MD, and the NIH Director's Challenge Award (PI: D. Levy). The analytical component of this project was funded by the Division of Intramural Research, National Heart, Lung, and Blood Institute, and the Center for Information Technology, National Institutes of Health, Bethesda, MD, and by the National Institute of Health grant U01 DK078616 and K24 DK080140 (PI: J.B. Meigs).
GENOA: Support for the Genetic Epidemiology Network of Arteriopathy (GENOA) was provided by the National Heart, Lung and Blood Institute (HL054457, HL100185, HL119443, HL133221) of the National Institutes of Health. We appreciate technical assistance from Jodie L. Van de Rostyne, Pamela I. Hammond, Julie M. Cunningham, and the Mayo Clinic Advanced Genomics Technology Center. We would also like to thank the families that participated in the GENOA study.
GOLDN and **HyperGEN**: This work was funded by National Heart, Lung, and Blood Institute grant U01-HL072524-04. B.H. was also funded by the National Heart, Lung, and Blood Institute, University of Alabama at Birmingham Statistical Genetics Postdoctoral Training Program, Grant 5T32-HL072757. Dr. Hidalgo was supported by an NHLBI Diversity Supplement (HHSN268201300001) and now supported by NHLBI K01 award (K01 HL130609-02), NHLBI PRIDE RISE (R25HL126146).
InCHIANTI: The InCHIANTI study baseline (1998-2000) was supported as a "Targeted Project" (ICS110.1/RF97.71) by the Italian Ministry of Health and in part by the U.S. National Institute on Aging (Contracts: 263 MD 9164 and 263 MD 821336). Analytic work described here was supported by the Intramural Research Program of the NIH, National Institute on Aging.
KORA: The KORA research platform (KORA, Cooperative Research in the Region of Augsburg) was initiated and financed by the Helmholtz Zentrum München-German Research Center for Environmental Health, Neuherberg, Germany and supported by grants from the German Federal Ministry of Education and Research (BMBF), the Federal Ministry of Health (Berlin, Germany), the Ministry of Innovation, Science, Research and Technology of the state North Rhine-Westphalia

(Düsseldorf, Germany), and the Munich Center of Health Sciences (MC Health) as part of LMUinnovativ. The German Diabetes Center is funded by the German Federal Ministry of Health (BMG) and the Ministry of Culture and Science of the State North Rhine-Westphalia. This study was also supported in part by a grant from the German Federal Ministry of Education and Research (BMBF) to the German Center for Diabetes Research (DZD e.V.).
WHI-BAA23: The Women's Health Initiative (WHI) was funded by the NHLBI through contracts HHSN268201100046C, HHSN268201100001C, HHSN268201100002C, HHSN268201100003C, HHSN268201100004C, and HHSN271201100004C. All contributors to WHI science are listed at https://www.whi.org/researchers/Documents%20%20Write%20a%20Paper/WHI%20Investigator%20Long%20List.pdf. Within WHI, "Epigenetic Mechanisms of PM-Mediated CVD Risk" (WHI-EMPC) was funded by NIEHS R01ES020836 (Whitsel; Baccarelli; Hou); "Integrative Genomics for Risk of CHD and Related Phenotypes in WHI" (WHI BAA23) by NHLBI 60442456 (Horvath; Assimes; Absher); "SNP Health Association Resource" project (WHI-SHARe) by NHLBI N02HL64278, "GWAS of Hormone Treatment and CVD and Metabolic Outcomes within the Genomics and Randomized Trials Network" (WHI-GARNET) by NHGRI U01HG005152; "GWAS to Identify Genetic Components of Hip Fracture" (WHI-HipFx) by NHLBI N01WH74316; and "Genetics and Epidemiology of Colorectal Cancer Consortium" (WHI-GECCO) by NCI U01CA137088 and R01CA059045. This work was additionally supported by R01HL129132 and R01HG006292 (Li).
WHI-EMPC: The Women's Health Initiative Epigenetic Mechanisms of PM-Mediated CVD (WHI-EMPC) is funded by the National Institute of Environmental Health Sciences (R01-ES020836; Whitsel, Baccarelli, Hou). The WHI program is funded by the National Heart, Lung, and Blood Institute, National Institutes of Health, U.S. Department of Health and Human Services through contracts HHSN268201100046C, HHSN268201100001C, HHSN268201100002C, HHSN268201100003C, HHSN268201100004C, HHSN271201100004C, HHSN268201600018C, HHSN268201600001C, HHSN268201600002C, HHSN268201600003C, and HHSN268201600004C. The authors thank the WHI investigators and staff for their dedication, and the study participants for making the program possible. A full listing of WHI investigators can be found at: http://www.whi.org/researchers/Documents%20%20Write%20a%20Paper/WHI%20Investigator%20Long%20List.pdf.

Supplementary Tables

Supplementary Table 1 Epigenome-wide association study (EWAS) results: known differentially methylated sites associated with fasting glucose or insulin at epigenome-wide significance level in the discovery phase

Locus	CpG	Chr	Position	Regulatory feature	Trait (s)	Model 1		Model 2		Previous evidence
						Beta	P-value*	Beta	P-value*	
DHCR24	cg17901584	1	55353706	Promoter associated (Cell type specific)	Insulin	-1.30	2.3×10^{-8}	-0.68	3.7×10^{-4}	BMI[1]; Insulin[1]; HbA1c[1]; Incident T2D[1]
4p15.33	cg10438589	4	14531493	NA	Insulin	1.25	2.4×10^{-8}	0.64	5.0×10^{-4}	BMI[1]; Insulin[1]; Incident T2D[1]
SLC7A11	cg06690548	4	139162808	NA	Glucose<br>Insulin	-1.98<br>-1.74	7.6×10^{-10}<br>2.4×10^{-8}	-1.58<br>-0.92	7.8×10^{-8}<br>2.8×10^{-4}	BMI[1]; Glucose[1]; Insulin[1]; Incident T2D[1]
RNF145	cg26403843	5	158634085	NA	Insulin	1.07	8.5×10^{-9}	0.69	7.7×10^{-6}	BMI[1, 2, 3]; Insulin[1]; Incident T2D[1]
CPT1A	cg00574958	11	68607622	NA	Glucose<br>Insulin	-2.67<br>-3.12	2.9×10^{-11}<br>3.5×10^{-13}	-1.89<br>-1.44	2.5×10^{-7}<br>3.5×10^{-5}	BMI[1, 2, 3, 4]; Glucose[1, 5]; Insulin[1]; HbA1c[1]; Incident T2D[1]; Prevalent T2D[2]
CPT1A	cg17058475	11	68607737	NA	Insulin	-1.78	7.4×10^{-9}	-1.04	3.4×10^{-5}	BMI[1]; Glucose[1]; Insulin[1]; HbA1c[1]; Incident T2D[1]
ASAM	cg26894079	11	122954435	NA	Insulin	-1.34	7.5×10^{-8}	-0.59	3.3×10^{-3}	BMI[1]; Insulin[1]; Incident T2D[1]
KDM2B	cg13708645	12	121974305	Promoter associated	Insulin	1.14	1.1×10^{-7}	0.59	8.8×10^{-4}	BMI[2, 3]; HbA1c[6]
MYO5C	cg06192883	15	52554171	Unclassified	Insulin	1.99	6.4×10^{-13}	1.05	4.4×10^{-6}	BMI[1, 2, 3]; Insulin[1]; Incident T2D[1]
SREBF1	cg11024682	17	17730094	Unclassified (Cell type specific)	Glucose<br>Insulin	1.87<br>2.07	2.7×10^{-8}<br>6.7×10^{-9}	1.26<br>0.78	6.4×10^{-5}<br>8.6×10^{-3}	BMI[1, 3, 7]; Glucose[1, 5]; Insulin[1]; HbA1c[1]; Incident T2D[1, 8]; Prevalent T2D[2, 9]
TMEM49	cg24174557	17	57903544	NA	Insulin	-1.59	8.8×10^{-12}	-0.76	6.7×10^{-5}	BMI[1, 2]; Insulin [1]; Incident T2D[1]
ABCG1	cg27243685	21	43642366	NA	Insulin	3.32	5.9×10^{-12}	2.04	4.5×10^{-7}	BMI[1, 2, 3, 7, 10]; Glucose[1]; Insulin[1]; HbA1c[1]; Incident T2D[1]
ABCG1	cg06500161	21	43656587	NA	Insulin	3.15	$< 2.2 \times 10^{-16}$	1.87	5.0×10^{-11}	BMI[1, 2, 3, 11]; Glucose[1, 5]; Insulin[1, 5, 6]; HbA1c[1]; Incident T2D[1, 8]; Prevalent T2D[2, 9, 12]; 2h glucose[5]; HOMA-IR[6]

Genome-wide CpG sites were tested for association with fasting glucose or fasting insulin in two models. The results of the previous reported CpG sites are shown. Model 1 adjusted for age, sex, technical covariates, white blood cell, and smoking status, accounting for family structure if needed in each cohort. Model 2 adjusted for body mass index (BMI) additionally. Locus: the cytogenetic location or the gene symbol of the CpG sites from Illumina annotation. Regulatory feature: the regulatory feature group of the CpG sites from Illumina annotation. Chr: Chromosome. Beta: effect estimate. * Genomic controlled P-value. Beta: effect estimate. NA: Not available. HbA1c: hemoglobin A1c. HOMA-IR: homeostatic model assessment-insulin resistance.

Supplementary Table 2 Blood-based expression quantitative trait methylations (eQTMs): association between gene expression and the glycemic CpG sites in blood

Locus (CpG)	CpG	Probe	Probe-Chr	Probe-Pos	Gene expression	Z	P-value	*Cis*/*Trans*
FCRL6	cg00936728	ENSG00000181036	1	159770301	*FCRL6*	-13.09	4.0×10^{-39}	*Cis*
SLAMF1	cg18881723	ENSG00000026751	1	160709037	*SLAMF7*	5.84	5.4×10^{-9}	*Cis*
SLAMF1	cg18881723	ENSG00000122223	1	160832692	*CD244*	4.68	2.9×10^{-6}	*Cis*
SLAMF1	cg18881723	ENSG00000117090	1	160617085	*SLAMF1*	-4.10	4.1×10^{-5}	*Cis*
CPT1A	cg00574958	ENSG00000110090	11	68611878	*CPT1A*	-9.22	3.1×10^{-20}	*Cis*
SREBF1	cg11024682	ENSG00000072310	17	17740325	*SREBF1*	-7.84	4.5×10^{-15}	*Cis*
ABCG1	cg06500161	ENSG00000160179	21	43619799	*ABCG1*	-12.78	2.2×10^{-37}	*Cis*

The gene expressions associated with the glycemic CpG sites are shown based on the European blood-based BIOS database (n = 3,841)[13]. Locus (CpG): the cytogenetic location or the gene symbol of the CpG sites from Illumina annotation. Probe: The probe of the gene expression. Chr: chromosome. Pos: position. Z: effect estimate per standard error.

Supplementary Table 3 Association between the gene expression level in the glucose metabolism-related tissues and the type 2 diabetes (T2D) or related traits based on MetaXcan database

Gene expression	Trait	Tissue	Z	P-value
FCRL6	T2D[14]	Liver	2.14	0.032
SREBF1	T2D[14]	Whole blood	-2.40	0.016
SREBF1	HbA1c[15]	Whole blood	-3.26	1.1×10^{-3}
KDM2B	FG	Skeletal muscle	2.5	0.011
MYO5C	FG	Pancreas	-4.5	6.0×10^{-6}
RBM20	FG	Pancreas	-2.4	0.015
MAN2A2	FG	Skeletal muscle	-2.3	0.023
APOBEC3H	HOMA-IR	Whole blood	2.6	0.010

The significant associations between the gene expression levels in the glucose metabolism-related tissue and the T2D or related traits are shown based on the MetaXcan database[16]. Only the effect direction of the association between the CpG sites, tissue-specific gene expression and traits were checked and only consistent effects were included in the table. It was explored in seven glucose-related tissues, i.e. adipose subcutaneous, adipose visceral omentum, liver, whole blood, pancreas, skeletal muscle and small intestine terminal ileum, and five T2D or related traits, i.e. T2D[14], fasting glucose (FG)[17, 18], fasting insulin (FI)[18], hemoglobin A1c (HbA1c)[19], and homeostatic model assessment-insulin resistance (HOMA-IR)[15]. Z: effect estimate per standard error.

Supplementary Table 4 Shared genetic determinants of glycemic CpG sites and type 2 diabetes (T2D) or related traits

Variant	Locus (meQTL)	Type (meQTL)	Chr	Position	MAF	EA	Association with CpG				Association with T2D or related traits†		
							CpG	Locus (CpG)	Z	P-value	Trait	Z	P-value
rs6701489	*TMEM61* (Nearest)	Protein coding	1	55358459	0.07	T	cg17901584	*DHCR24*	4.82	1.4×10^{-6}	FG[18]	-3.43	8.5×10^{-4}
rs6896438	*RNF145* (Nearest)	Protein coding	5	158547876	0.36	C	cg26403843	*RNF145*	6.15	8.0×10^{-10}	FI[17]	3.82	1.4×10^{-4}
rs10849885	*KDM2B*	Protein coding	12	121881848	0.32	A	cg13708645	*KDM2B*	29.33	4.2×10^{-189}	FG[18]	4.17	2.2×10^{-5}
rs9374080	*CCDC162P*	pseudogene	6	109616420	0.27	C	cg20507228	*MAN2A2*	-5.31	1.1×10^{-7}	HbA1c[20]	-5.11	2.0×10^{-7}
rs3818717*	*RAI1*	Protein coding	17	17707105	0.06	T	cg11024682	*SREBF1*	8.93	4.1×10^{-19}	T2D[21]	1.08	4.9×10^{-4}
rs7529925	*RP11-16L9.4*	lincRNA	1	199007208	0.21	C	cg11024682	*SREBF1*	-5.15	2.6×10^{-7}	HbA1c[20]	-3.60	2.5×10^{-4}
rs16960744*	*TOM1L2*	Protein coding	17	17755259	0.37	A	cg11024682	*SREBF1*	4.87	1.1×10^{-6}	HbA1c[20]	3.11	1.5×10^{-3}

The table shows the shared genetic determinants (SNP level) of glycemic CpG sites and T2D or related traits. The direction of effect estimates for effect alleles of Methylation quantitative trait loci (meQTL) associations and T2D-related trait associations were checked and only consistent effects were included in the table. Chr: chromosome. Locus (meQTL): the located or nearest protein-coding gene of the meQTL from UCSC annotation. Type (meQTL): the gene type of the meQTL. MAF: minor allele frequency. EA: effect allele. Locus (CpG): the cytogenetic location or the gene symbol of the CpG sites from Illumina annotation. Z: effect estimate per standard error. FG: fasting glucose. FI: fasting insulin. Data sources of associations: 1) association with CpG was from the current discovery phase (n = 4,808), 2) associations with FG (n = 133,010), FI (n = 96,496), T2D (case/control: n = 81,412/370,832) and hemoglobin A1c (HbA1c; n = 159,940) were from public GWAS database[17, 18, 20, 21]. * SNPs in linkage disequilibrium (LD): R^2 (rs3818717 and rs16960744) = 0.61.

Supplementary Table 5 Shared genetic determinants of glycemic DNA methylation (methylation quantitative trait loci, meQTL) and gene expression (expression quantitative trait loci, eQTL) in blood.

							Association with CpG					Association with gene expression			
**Variant**	**Chr**	**Position**	**Locus (eQTL)**	**Type (eQTL)**	**MAF**	**EA**	**CpG**	**Locus (CpG)**	**Z**	**P-value**	***Cis*/*Trans***	**Gene expression**	**Z**	**P-value**	***Cis*/*Trans***
rs11265282*	1	159774408	*FCRL6*	Protein coding	0.26	C	cg00936728	*FCRL6*	4.17	3.0×10^{-5}	*Cis*	*FCRL6*	-6.73	1.7×10^{-11}	*Cis*
rs1577544*	1	160630974	*SLAMF1* (Nearest)	Protein coding	0.39	T	cg18881723	*SLAMF1*	-5.45	5.1×10^{-8}	*Cis*	*SLAMF1*	-6.40	1.6×10^{-10}	*Cis*
rs6502629	17	17869642	*TOM1L2*	Protein coding	0.22	G	cg11024682	*SREBF1*	9.97	2.1×10^{-23}	*Cis*	*SREBF1*	-17.93	7.2×10^{-72}	*Cis*

The shared genetic determinants (SNP level) of glycemic CpG sites and gene expression that is glycemic CpG sites related in the blood-based BIOS database (n = 3,841) [13] are shown. * SNP in linkage disequilibrium (LD) with the top SNP in **Supplementary Data 3**: R^2 (rs11265282 and rs6657365) = 0.96, R^2 (rs1577544 and rs11265461) = 0.56. Locus (eQTL): the located or nearest protein-coding gene of the eQTL. Type (eQTL): the gene type of the eQTL. Chr: chromosome. MAF: minor allele frequency. EA: effect allele. Z: effect estimate per standard error. Locus (CpG): the cytogenetic location or the gene symbol of the CpG sites from Illumina annotation.

Supplementary Table 6 Correlation between the nine novel CpGs

cpg	cg00936728	cg13222915	cg18881723	cg13729116	cg15880704	cg25924746	cg20507228	cg06229674	cg18247172
cg00936728.r	1.00	0.02	-0.57	-0.42	-0.05	-0.45	-0.33	0.65	0.63
cg00936728.p	0	0.44	3.9×10^{-134}	1.4×10^{-68}	0.051	3.6×10^{-78}	4.6×10^{-41}	9.9×10^{-187}	8.8×10^{-174}
cg13222915.r	0.02	1.00	0.19	-0.19	0.11	0.40	0.52	-0.08	-0.16
cg13222915.p	0.44	0	2.8×10^{-14}	2.3×10^{-13}	3.0×10^{-5}	1.4×10^{-59}	1.3×10^{-109}	1.6×10^{-3}	2.1×10^{-10}
cg18881723.r	-0.57	0.19	1.00	0.05	0.03	0.32	0.39	-0.60	-0.70
cg18881723.p	3.9×10^{-134}	2.8×10^{-14}	0	0.055	0.300	5.1×10^{-39}	2.4×10^{-56}	8.7×10^{-152}	4.5×10^{-227}
cg13729116.r	-0.42	-0.19	0.05	1.00	0.05	0.38	0.04	-0.16	-0.25
cg13729116.p	1.4×10^{-68}	2.3×10^{-13}	0.055	0	0.051	3.6×10^{-54}	0.10	5.6×10^{-10}	1.7×10^{-23}
cg15880704.r	-0.05	0.11	0.03	0.05	1.00	0.31	0.34	0.04	0.07
cg15880704.p	0.051	3.0×10^{-5}	0.300	0.051	0	1.6×10^{-35}	9.8×10^{-44}	0.12	0.100
cg25924746.r	-0.45	0.40	0.32	0.38	0.31	1.00	0.65	-0.32	-0.33
cg25924746.p	3.6×10^{-78}	1.4×10^{-59}	5.1×10^{-39}	3.6×10^{-54}	1.6×10^{-35}	0	7.7×10^{-183}	4.6×10^{-37}	1.4×10^{-39}
cg20507228.r	-0.33	0.52	0.39	0.04	0.34	0.65	1.00	-0.37	-0.31
cg20507228.p	4.6×10^{-41}	1.3×10^{-109}	2.4×10^{-56}	0.100	9.8×10^{-44}	7.7×10^{-183}	0	1.6×10^{-52}	9.3×10^{-37}
cg06229674.r	0.65	-0.08	-0.60	-0.16	0.04	-0.32	-0.37	1.00	0.69
cg06229674.p	9.9×10^{-187}	1.6×10^{-3}	8.7×10^{-152}	5.6×10^{-10}	0.120	4.6×10^{-37}	1.6×10^{-52}	0	3.3×10^{-219}
cg18247172.r	0.63	-0.16	-0.70	-0.25	0.07	-0.33	-0.31	0.69	1.00
cg18247172.p	8.8×10^{-174}	2.1×10^{-10}	4.5×10^{-227}	1.7×10^{-23}	0.010	1.4×10^{-39}	9.3×10^{-37}	3.3×10^{-219}	0

The correlation of the novel CpG sites was checked by Pearson's correlation test (n = 1,544). .r: correlation coefficient. .p: P-value from the Pearson’s correlation test.

Supplementary Table 7 The enriched biological processes of genes from new glycemic methylation loci

GO biological process complete (Gene Sets)	Number of genes in the database	Number of observed genes in the testing set	P-value	P-value (FDR)	Observed genes
Positive regulation of leukocyte proliferation	135	2	1.1×10^{-5}	5.0×10^{-3}	*SLAMF1, IRS2*
Hexose metabolic process	157	2	1.8×10^{-5}	5.0×10^{-3}	*IRS2, MAN2A2*
Monosaccharide metabolic process	202	2	3.9×10^{-5}	5.2×10^{-3}	*IRS2, MAN2A2*
Regulation of leukocyte proliferation	203	2	3.9×10^{-5}	5.2×10^{-3}	*SLAMF1, IRS2*
Positive regulation of protein secretion	209	2	4.3×10^{-5}	5.3×10^{-3}	*SLAMF1, IRS2*
Positive regulation of cell activation	285	2	1.1×10^{-4}	8.2×10^{-3}	*SLAMF1, IRS2*
Positive regulation of secretion	368	2	2.3×10^{-4}	0.013	*SLAMF1, IRS2*
Regulation of protein secretion	386	2	2.6×10^{-4}	0.014	*SLAMF1, IRS2*
Immune effector process	454	2	4.2×10^{-4}	0.019	*SLAMF1, APOBEC3H*
Negative regulation of transport	455	2	4.3×10^{-4}	0.019	*SLAMF1, IRS2*
Regulation of cell activation	458	2	4.3×10^{-4}	0.019	*SLAMF1, IRS2*
Positive regulation of establishment of protein localization	512	2	6.0×10^{-4}	0.023	*SLAMF1, IRS2*
Innate immune response	587	2	9.0×10^{-4}	0.030	*SLAMF1, APOBEC3H*
Carbohydrate metabolic process	652	2	1.2×10^{-3}	0.038	*IRS2, MAN2A2*
Cell proliferation	667	2	1.3×10^{-3}	0.040	*SLAMF1, IRS2*
Regulation of secretion	694	2	1.5×10^{-3}	0.043	*SLAMF1, IRS2*

Gene ontology (GO) biological process[22, 23, 24] was obtained from Molecular Signatures Database (MsigDB) c5[25]. FDR: false discovery rate.

References

1. Wahl S, *et al.* Epigenome-wide association study of body mass index, and the adverse outcomes of adiposity. *Nature* **541**, 81-86 (2017).
2. Al Muftah WA, *et al.* Epigenetic associations of type 2 diabetes and BMI in an Arab population. *Clin Epigenetics* **8**, 13 (2016).
3. Demerath EW, *et al.* Epigenome-wide association study (EWAS) of BMI, BMI change and waist circumference in African American adults identifies multiple replicated loci. *Hum Mol Genet* **24**, 4464-4479 (2015).
4. Aslibekyan S, *et al.* Epigenome-wide study identifies novel methylation loci associated with body mass index and waist circumference. *Obesity (Silver Spring)* **23**, 1493-1501 (2015).
5. Kriebel J, *et al.* Association between DNA Methylation in Whole Blood and Measures of Glucose Metabolism: KORA F4 Study. *PLoS One* **11**, e0152314 (2016).
6. Hidalgo B, *et al.* Epigenome-wide association study of fasting measures of glucose, insulin, and HOMA-IR in the Genetics of Lipid Lowering Drugs and Diet Network study. *Diabetes* **63**, 801-807 (2014).
7. Wang B, *et al.* Methylation loci associated with body mass index, waist circumference, and waist-to-hip ratio in Chinese adults: an epigenome-wide analysis. *Lancet* **388 Suppl 1**, S21 (2016).
8. Chambers JC, *et al.* Epigenome-wide association of DNA methylation markers in peripheral blood from Indian Asians and Europeans with incident type 2 diabetes: a nested case-control study. *Lancet Diabetes Endocrinol* **3**, 526-534 (2015).
9. Walaszczyk E, *et al.* DNA methylation markers associated with type 2 diabetes, fasting glucose and HbA1c levels: a systematic review and replication in a case-control sample of the Lifelines study. *Diabetologia* **61**, 354-368 (2018).
10. Wilson LE, Harlid S, Xu Z, Sandler DP, Taylor JA. An epigenome-wide study of body mass index and DNA methylation in blood using participants from the Sister Study cohort. *Int J Obes (Lond)* **41**, 194-199 (2017).
11. Ding J, *et al.* Alterations of a Cellular Cholesterol Metabolism Network Are a Molecular Feature of Obesity-Related Type 2 Diabetes and Cardiovascular Disease. *Diabetes* **64**, 3464-3474 (2015).
12. Kulkarni H, *et al.* Novel epigenetic determinants of type 2 diabetes in Mexican-American families. *Hum Mol Genet* **24**, 5330-5344 (2015).
13. Bonder MJ, *et al.* Disease variants alter transcription factor levels and methylation of their binding sites. *Nat Genet* **49**, 131-138 (2017).
14. Replication DIG, *et al.* Genome-wide trans-ancestry meta-analysis provides insight into the genetic architecture of type 2 diabetes susceptibility. *Nat Genet* **46**, 234-244 (2014).
15. Dupuis J, *et al.* New genetic loci implicated in fasting glucose homeostasis and their impact on type 2 diabetes risk. *Nat Genet* **42**, 105-116 (2010).
16. Barbeira AN, *et al.* Exploring the phenotypic consequences of tissue specific gene expression variation inferred from GWAS summary statistics. *Nat Commun* **9**, 1825 (2018).

17. Manning AK*, et al.* A genome-wide approach accounting for body mass index identifies genetic variants influencing fasting glycemic traits and insulin resistance. *Nat Genet* **44**, 659-669 (2012).

18. Scott RA*, et al.* Large-scale association analyses identify new loci influencing glycemic traits and provide insight into the underlying biological pathways. *Nat Genet* **44**, 991-1005 (2012).

19. Soranzo N*, et al.* Common variants at 10 genomic loci influence hemoglobin A(1)(C) levels via glycemic and nonglycemic pathways. *Diabetes* **59**, 3229-3239 (2010).

20. Wheeler E*, et al.* Impact of common genetic determinants of Hemoglobin A1c on type 2 diabetes risk and diagnosis in ancestrally diverse populations: A transethnic genome-wide meta-analysis. *PLoS Med* **14**, e1002383 (2017).

21. Mahajan A*, et al.* Refining the accuracy of validated target identification through coding variant fine-mapping in type 2 diabetes. *Nat Genet* **50**, 559-571 (2018).

22. Mi H*, et al.* PANTHER version 11: expanded annotation data from Gene Ontology and Reactome pathways, and data analysis tool enhancements. *Nucleic Acids Res* **45**, D183-D189 (2017).

23. Ashburner M*, et al.* Gene ontology: tool for the unification of biology. The Gene Ontology Consortium. *Nat Genet* **25**, 25-29 (2000).

24. The Gene Ontology C. Expansion of the Gene Ontology knowledgebase and resources. *Nucleic Acids Res* **45**, D331-D338 (2017).

25. Subramanian A*, et al.* Gene set enrichment analysis: a knowledge-based approach for interpreting genome-wide expression profiles. *Proc Natl Acad Sci USA* **102**, 15545-15550 (2005).

Supplementary Figures

a

b

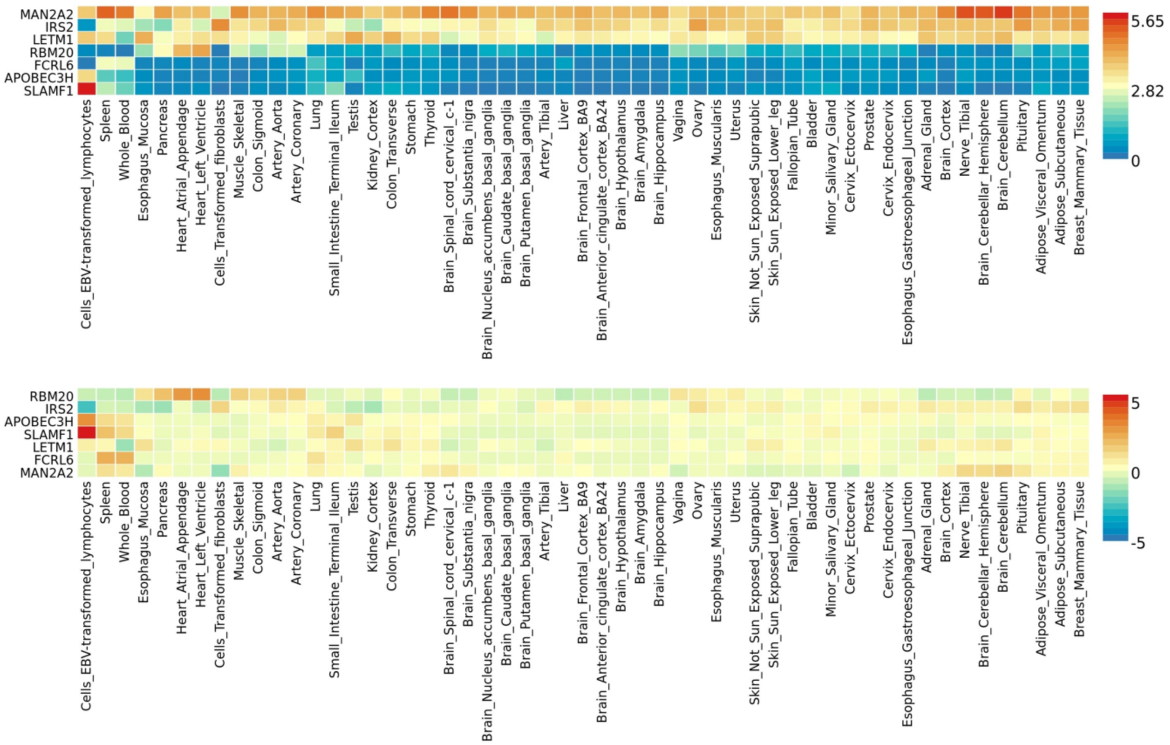

Supplementary Figure 1 Tissue-specific expression patterns from 53 Genotype-Tissue Expression (GTEx) tissues for the novel genes from epigenome-wide association study (EWAS)

The genes and tissues are ordered by the clusters. Seven of the nine novel CpG sites were annotated to genes and shown, and other two were located in the non-coding region. In Figure 1a, this is an average of log2 transformed per label. In Darker red means a higher expression of that gene, compared to a darker blue color. Figure 1b, the average value of the relative expression value (zero mean normalization of log2 transformed expression. Darker red means a higher relative expression of that gene, compared to a darker blue color.

a

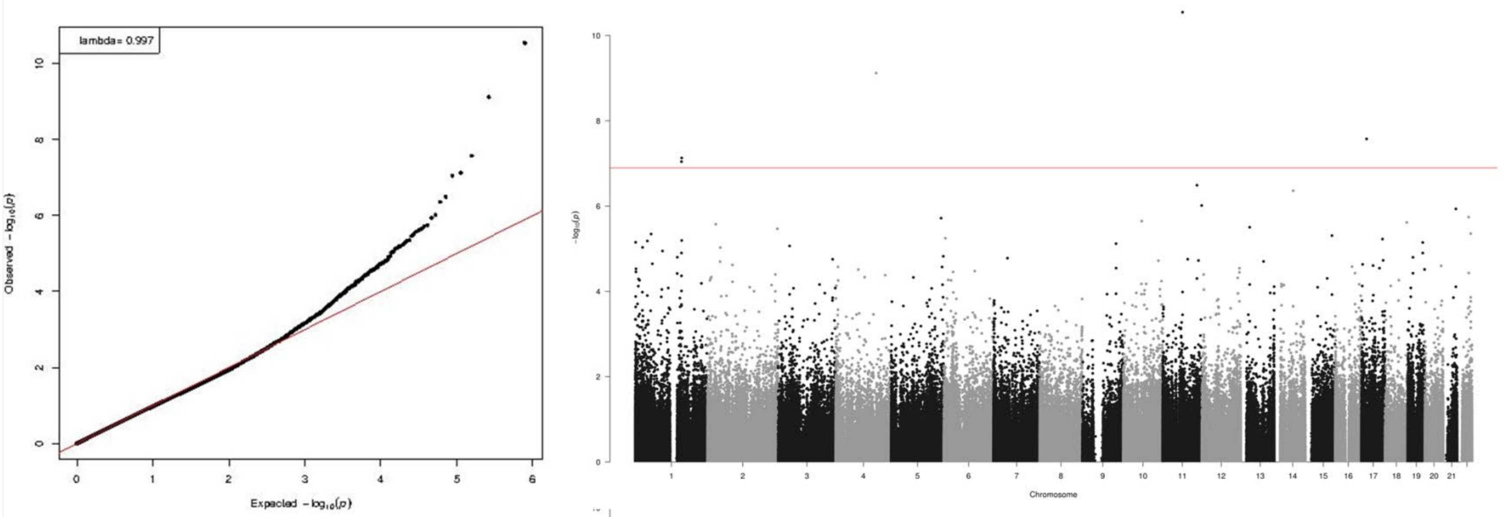
lambda= 0.997
Observed -log10(p)
Expected -log10(p)
-log10(p)
Chromosome

b

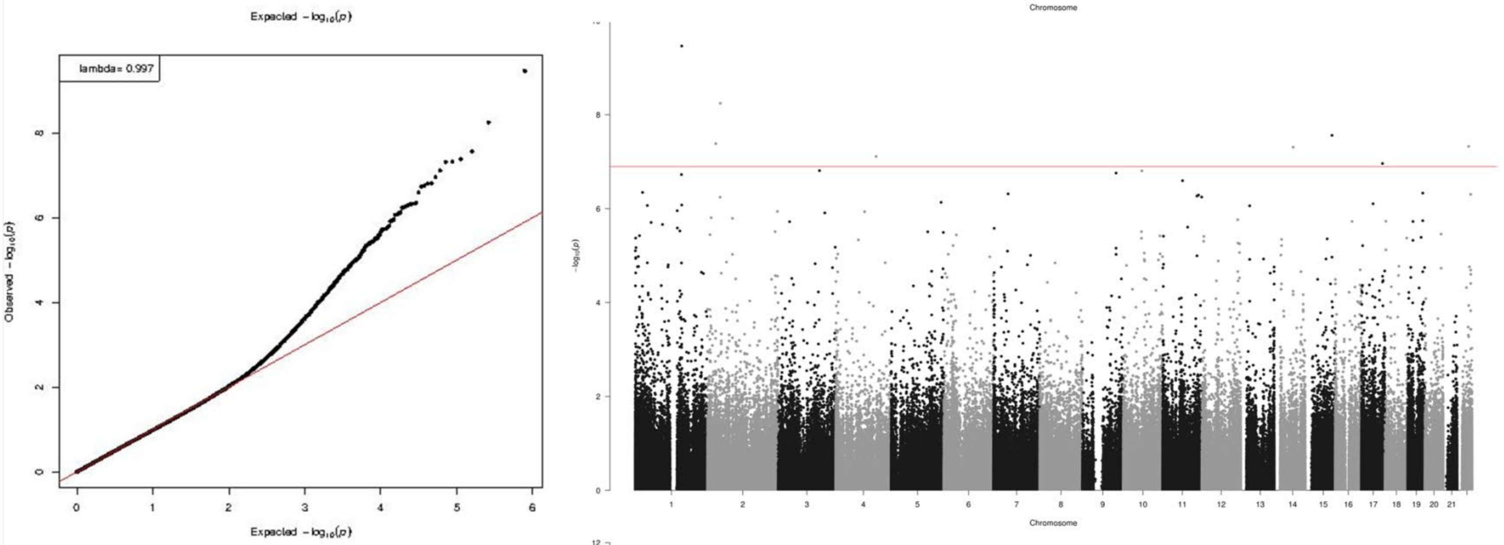
lambda= 0.997
Observed -log10(p)
Expected -log10(p)
-log10(p)
Chromosome

c

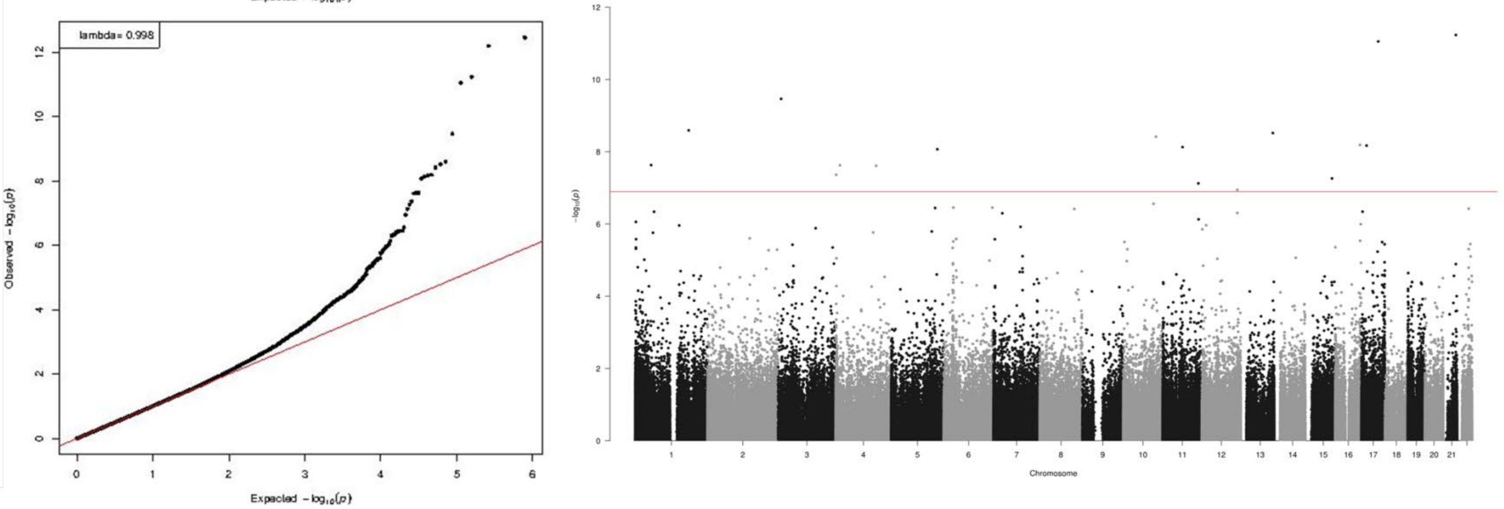
lambda= 0.998
Observed -log10(p)
Expected -log10(p)
-log10(p)
Chromosome

d

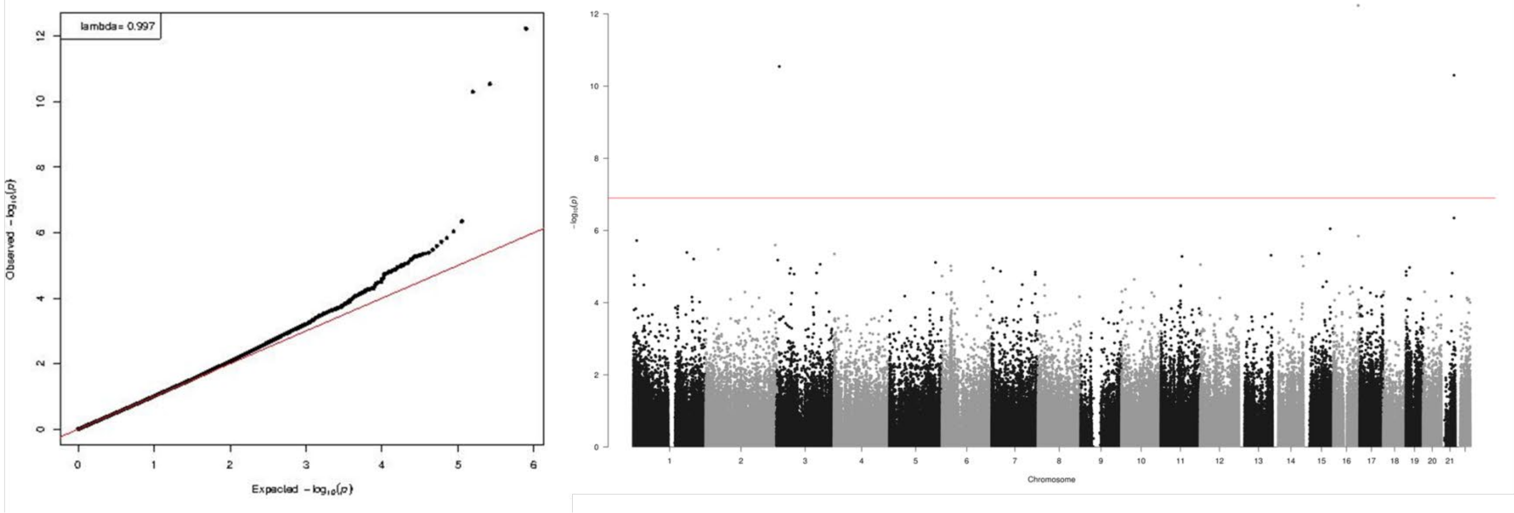
lambda= 0.997
Observed -log10(p)
Expected -log10(p)
-log10(p)
Chromosome

Supplementary Figure 2 Quantile-quantile (QQ) plots and Manhattan plots of the epigenome-wide association study (EWAS) results

A: EWAS results of fasting glucose in the baseline model; B: EWAS results of fasting glucose in the body mass index (BMI)-adjusted model; C: EWAS results of fasting insulin in the baseline model; D: D EWAS results of fasting insulin in the BMI-adjusted model. Source data are provided as a Source Data file.

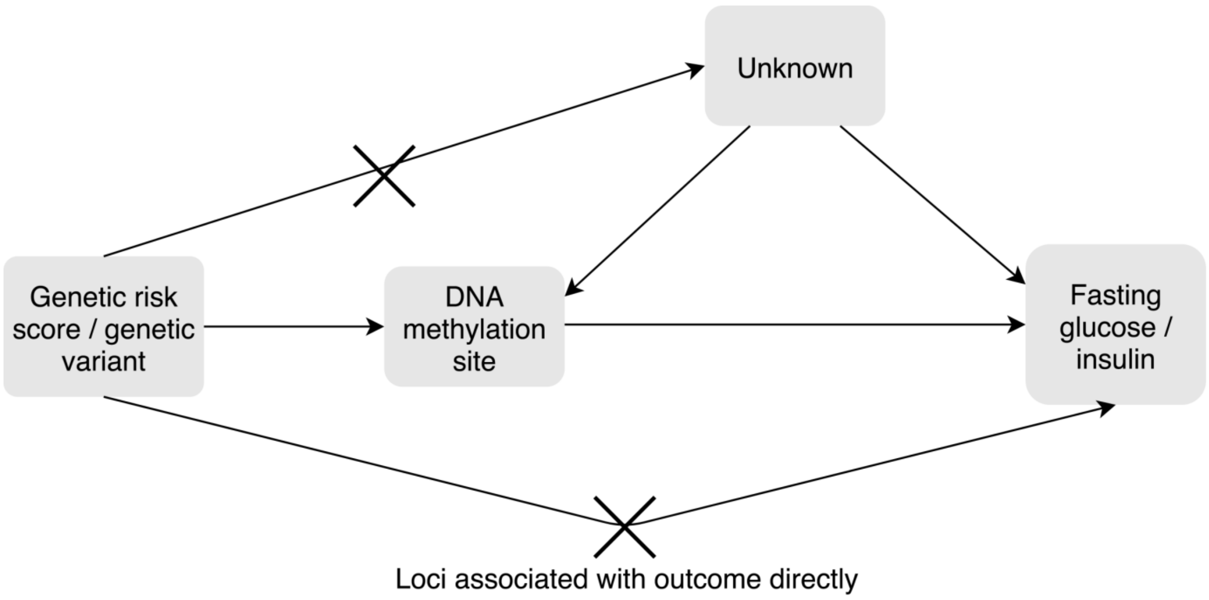

Supplementary Figure 3 Overview of the general Mendelian Randomization process
