# Supplementary material for: An integrative cross-omics analysis of DNA methylation sites of glucose and insulin homeostasis
Source: Nat Commun. 2019 Jun 13;10:2581. doi: 10.1038/s41467-019-10487-4 (PMC6565679; doi:10.1038/s41467-019-10487-4)
Supplement: Supplementary file 3 — Description of Additional Supplementary Files [file 41467_2019_10487_MOESM3_ESM.pdf]

Description of Additional Supplementary Files

Supplementary Data 1. Characteristics of the cohorts

The units of fasting insulin in FHS, GOLDN, HyperGEN, WHI-BAA23 are mIU/L; the units of fasting insulin in other cohorts are pmol/L. EA: European ancestry. AA: African ancestry. HA: Hispanic ancestry. NA: Not available.

Supplementary Data 2. Epigenome-wide association study (EWAS) results: replication of newly discovered differentially methylated sites in different ancestry populations.

Replication of the epigenome-wide significant (P-value < 1.27 × 10-7) CpG sites with fasting glucose or insulin stratified by different ancestry populations. Model 1 adjusted for age, sex, technical covariates, white blood cell, and smoking status, accounting for family structure if needed in each cohort. Model 2 adjusted for body mass index (BMI) additionally. EA: European ancestry (n = 6,778 for fasting glucose; n = 6,773 for fasting insulin). AA: African ancestry (n = 4,355 for fasting glucose; n = 2,434 for fasting insulin). HA: Hispanic ancestry (n = 577 for fasting glucose; n = 560 for fasting insulin). NP: Replication was not performed in the non-significant associated model or trait from the discovery phase. Locus: the cytogenetic location or the gene symbol of the CpG sites from Illumina annotation. Chr: Chromosome. Beta: effect estimate. The beta in the replication phase was from the model with fasting glucose or log-transformed fasting insulin as the independent variable and beta of methylation level as the dependent variable. Bold print: Bonferroni significant results (P-value < 3.3 × 10-3).

Supplementary Data 3. Methylation quantitative trait loci (meQTLs) for known or new replicated CpG sites.

Based on the blood-based BIOS database (n = 3,841), the meQTL information of known or new replicated CpG sites are shown. Locus (CpG): the cytogenetic location or the gene symbol of the CpG sites from Illumina annotation. Locus (meQTL): the located or nearest protein-coding gene of the meQTL from UCSC annotation. Type (meQTL): the gene type of the meQTL. Chr: chromosome. MAF: minor allele frequency. EA: effect allele. Z: effect estimate per standard error.

Supplementary Data 4. Mendelian randomization (MR) results.

Two-sample MR approach was performed to check the effect of known or replicated CpG sites on their significant traits, either fasting glucose (FG) or fasting insulin (FI). We also performed MR test in the cis-only SNPs if the CpG has both cis and trans genetic markers. Locus (CpG): the cytogenetic location or the gene symbol of the CpG sites from Illumina annotation. R2 (%): the percentage of explained variance in the exposure by genetic risk score. Effect/SE/P-value: The effect estimate / standard error / P-value of genetic risk score of the exposure on the outcome (MR results). Heterogeneity P-value: The P-value of the heterogeneity test among the SNPs. Type of SNPs: type of SNPs included in the genetic risk score: 1) CisTrans: all the genetic markers (included cis and trans SNPs); 2) Only-Cis: only cis genetic markers available; 3) Only-Trans: only trans genetic markers available; 4) Sub-Cis: the sub-analysis with the genetic markers in cis-only. Z (exposure): the effect estimate per standard error of the SNP on exposure (CpG) from exposure GWAS result; Z (outcome): the effect estimate per standard error of the SNPs on the outcome (fasting glucose or insulin) from outcome GWAS result. NP: the genetic risk score has R2 less than 1%, and the MR was not performed. NA: Not available.
